# Showcasing the optical properties of monocrystalline zinc phosphide thin films as an earth-abundant photovoltaic absorber†

**DOI:** 10.1039/d1ma00922b

**Published:** 2021-12-17

**Authors:** Elias Z. Stutz, Mahdi Zamani, Djamshid A. Damry, Léa Buswell, Rajrupa Paul, Simon Escobar Steinvall, Jean-Baptiste Leran, Jessica L. Boland, Mirjana Dimitrievska, Anna Fontcuberta i Morral

**Affiliations:** Laboratory of Semiconductor Materials, Institute of Materials, Faculty of Engineering, École Polytechnique Fédérale de Lausanne 1015 Lausanne Switzerland mirjana.dimitrievska@epfl.ch anna.fontcuberta-morral@epfl.ch; Photon Science Institute, Department of Electrical and Electronic Engineering, University of Manchester Alan Turing Building, Oxford Road Manchester M13 9PL UK; Institute of Physics, Faculty of Basic Sciences, École Polytechnique Fédérale de Lausanne 1015 Lausanne Switzerland

## Abstract

Zinc phosphide, Zn_3_P_2_, is a semiconductor with a high absorption coefficient in the spectral range relevant for single junction photovoltaic applications. It is made of elements abundant in the Earth's crust, opening up a pathway for large deployment of solar cell alternatives to the silicon market. Here we provide a thorough study of the optical properties of single crystalline Zn_3_P_2_ thin films grown on (100) InP by molecular beam epitaxy. The films are slightly phosphorus-rich as determined by Rutherford backscattering. We elucidate two main radiative recombination pathways: one transition at approximately 1.52 eV attributed to zone-center band-to-band electronic transitions; and a lower-energy transition observed at 1.3 eV to 1.4 eV attributed to a defect band or band tail related recombination mechanisms. We believe phosphorus interstitials are likely at the origin of this band.

## Abstract 2

Optoelectronic properties of zinc phosphide (Zn_3_P_2_), a promising photovoltaic absorber, are explored using photoluminescence and terahertz-probe spectroscopy.

## Graphical abstract

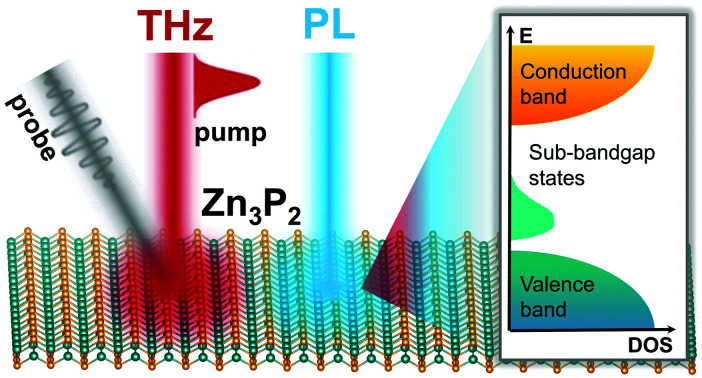

## 1. Introduction

Direct-bandgap solar absorbers with non-toxic components that are abundant in the Earth's crust are essential for replacing photovoltaic technologies using scarce and toxic materials. Earth-scarce indium, gallium, selenium or tellurium are all part of commercially-available CIGSSe (copper indium gallium sulfide selenide) and CdTe photovoltaic solar cells. Technologies based on these elements are precluded from ever reaching the terawatt energy production scales needed to sustainably supply renewable energy without using vanishingly small amounts of material.^1,2^ A semiconductor with non-toxic components satisfying both the needs for abundance and suitable properties for light absorption in photovoltaics is zinc phosphide, α-Zn_3_P_2_,^3^ hereafter referred to as Zn_3_P_2_. This semiconductor has a direct 1.5 eV bandgap, strongly absorbs visible light (>10^4^–10^5^ cm^−1^)^4,5^ and exhibits carrier diffusion lengths of several micrometers.^6^ However, two challenges have been holding back the power conversion efficiency of zinc phosphide solar cells, with the record standing at 6% for the past 40 years.^7^ First, Zn_3_P_2_ has a relatively large coefficient of thermal expansion (CTE), compared to conventional III–V semiconductors.^8^ Combined with the absence of commercially-available lattice-matched substrates, this can induce large thermal-mismatch strain during cooling after processing and the formation of mismatch dislocations and cracks. Several successful workarounds have been shown in the past years. For example, the growth of Zn_3_P_2_ on graphene completely removes the influence of lattice- and CTE-mismatch by removing the formation of covalent bonds at the interface.^9^ Whereas, the growth of nanostructures, either vapor–liquid–solid-grown nanowires^10^ or by selected area epitaxy limiting growth to nanoscale areas^11^ allows the formation of higher-quality crystals with their increased control of mismatch strain and defects close to the interface. The second main challenge in the development of zinc phosphide is doping. This semiconductor has almost always been found to be intrinsically p-doped, due to the small formation energy of intrinsic acceptor defects such as phosphorus interstitials.^12,13^ Doping engineering is critical for the formation of homo- and heterojunctions with properties tailored for photovoltaic applications and reaching the expectations of this promising PV material. Defects can also influence other electronic properties, such as carrier lifetimes,^14^ and can lead to localized bandgap fluctuations,^15^ all of which contribute to the efficiency of electronic devices.^16^ Therefore, a thorough understanding of the defects, their electronic properties and their interplay with the growth conditions is crucial for the development of photovoltaic cells with this earth-abundant absorber.

Photoluminescence (PL) spectroscopy can provide relevant information on the material properties. It is highly sensitive to the presence of defects and the electronic band structure, as they are often involved in radiative recombination processes.^17^ This non-destructive and contactless technique is commonly used to characterize the optoelectronic properties of semiconductors. The different characteristics of the PL peaks are related to the average properties of defects or electronic bands, such as their relative positions and their concentrations.^18,19^ Uncontrolled growth and poor crystalline quality can increase the complexity and variability of the PL spectra, making their interpretation more challenging and less reliable. To date, some of the most complete PL studies of this semiconductor were done by Briones *et al.*, who studied the details of pair transitions at low temperatures,^20^ and by Kimball *et al.*, investigating the PL emission of polycrystalline Zn_3_P_2_ wafers from 5.9 K to 310 K.^6^

In this work, we investigate the steady-state PL spectrum of monocrystalline zinc phosphide thin films grown by molecular beam epitaxy on one of the best lattice-matched substrates, (100) InP. Temperature-dependent measurements are complemented by power-dependent PL measurements to study the electronic transitions involved in the radiative emission pathways. We also characterize the carrier dynamics *via* another non-contact technique, optical-pump terahertz-probe (OPTP) spectroscopy and demonstrate long lifetimes suitable for photovoltaic applications. To the best of our knowledge, this is the most thorough description of the PL spectrum of a high-quality Zn_3_P_2_ thin film, and one of the most thorough PL studies of zinc phosphide overall. Our results can be used as a basis of comparison for the defect characterization of more complex and disordered Zn_3_P_2_ structures, leading to the characterization of the interplay between the processing conditions and optoelectronic properties of the semiconductor.

## 2. Experimental section

The thin films studied here were prepared following ref. 21. X-Ray diffraction measurements were performed using a Panalytical Empyrean diffractometer operating in Gonio scan configuration with a copper (K_α_) X-ray source with 1.54 Å wavelength, operating at 35 keV and 40 mA. The scanning electron microscopy images were taken in a Zeiss MERLIN™ field emission scanning electron microscope (SEM) with a 20° tilt and an Inlens detector. The micro-Raman spectrum has been acquired using a TriVista triple spectrometer with 900, 900 and 1800 mm^−1^ gratings in subtractive mode and a Princeton Instruments liquid-nitrogen-cooled multichannel CCD PyLoN camera. The excitation light was the 532 nm line of a Coherent Sapphire SF optically-pumped semiconductor laser and was focused onto the sample in back-scattering geometry with a microscope objective (numerical aperture: 0.75), reaching spot diameters of about 1 μm. A Gaussian filter with a standard deviation of 1.5 pixels (approximately 0.88 cm^−1^) was used to smooth the Raman spectrum. The measurement was carried out at 12 K in a cryostat pumped to roughly 5 × 10^−7^ mbar. The same cryostat and focusing objective were used to carry out the micro-photoluminescence measurements. Those were captured with an Andor iDus DV420A-OE detector, illuminated with the 488 nm line of a Coherent Sapphire laser. The photoluminescence spectra were corrected for the spectral sensitivity of the detection system and transformed to the energy scale with the Jacobian transformation. Parasitic features of the spectra, such as cosmic rays and a diffracted peak of the laser at 1.27 eV have been replaced with artificial data points for clarity and are shown as different color shades in the figures. Rutherford backscattering spectrometry measurements, carried out by EAG Laboratories, were taken with a nearly-normally-incident beam of 2.275 MeV alpha particles. The normal detector angle collected particles scattered by 160° and the grazing detector was set at 104°. Assumptions of 6.61 × 10^22^ atoms per cm^3^ in the zinc phosphide layer and 5.26 × 10^22^ atoms per cm^3^ in the indium phosphide were used, and the atomic concentration uncertainty is ±1%.

Optical-pump terahertz-probe spectroscopic measurements were carried out using a spectrometer based on an ultrafast Ti:Sapphire amplifier (Newport Spectra Physics Spitfire Ace, 13 mJ, 1 kHz, 40 fs) described in ref. 22. The terahertz probe was generated by optical rectification in a GaP crystal, before being focused onto the sample. The reflected THz beam was then detected by electro-optic sampling in ZnTe using a balanced photodiode scheme and a high-precision, high-resolution oscilloscope (Pico Technology PicoScope 4262) for data acquisition. To measure in reflection, a silver-coated prism was placed near the focus of the OPTP spectrometer. The THz beam reflected from one side of the prism, onto the sample, off the second face of the prism and subsequently continued along the initial THz beam axis. The angle of incidence was <15° from normal incidence. The sample was photoexcited with a pulse centered at 750 nm (*E*_photon_ = 1.65 eV) and pulse duration of 40 fs at fluences between 12 and 128 μJ cm^−2^. Photoexcitation at this wavelength generates electron–hole pairs within the Zn_3_P_2_ thin films modifying the dielectric landscape of the sample and inducing a change, Δ*E*, in the reflection of the electric field of the terahertz probe pulse, *E*. This value of Δ*E*/*E* is proportional to the photoconductivity of the sample and thus to the free carrier concentration.^23^ The temporal evolution of this response therefore provides information on the carrier lifetime and mobility of the sample.^24^

## 3. Results and discussion

### 3.1. Crystalline properties

**Fig. 1:**
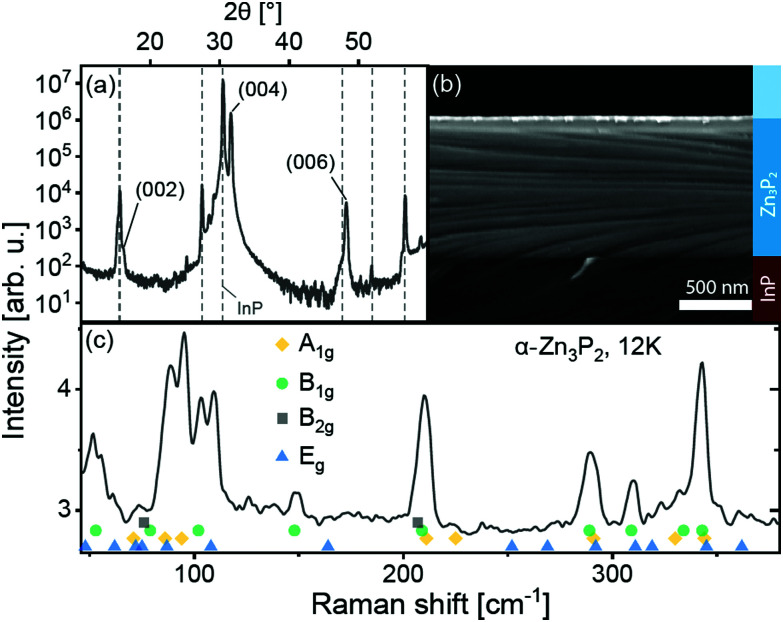
Representative (a) X-ray diffraction (XRD) spectrum, (b) tilted cross-section scanning electron micrograph and (c) Raman spectrum of a Zn_3_P_2_ thin film on InP. XRD peaks assigned to the InP substrate are shown with dashed lines. The experimentally-determined peak positions of the phonons of Zn_3_P_2_, from ref. 28, are shown with colored symbols.

First, the crystalline structure of the thin film is characterized. The X-ray diffraction pattern measured in gonio geometry from the thin film and substrate structure is shown in Fig. 1a. As elucidated in ref. 21, this pattern is a characteristic fingerprint of monocrystalline thin films on InP. The monocrystallinity of the film is also verified by transmission electron microscopy selected area diffraction patterns of thin film cross-sections in data presented in ref. 21 and 25. Fig. 1b shows a tilted scanning electron micrograph of a cleaved cross-section. The film surface is slightly undulated due to the uneven growth of a thin layer of native oxide.^26^ At about 800 nm thickness, the Zn_3_P_2_ film is thick enough to absorb most of the incident laser light. The 99% attenuation length in zinc phosphide for the light of the two lasers used for optical spectroscopy in this study is approximately 280 nm and 200 nm, for 532 nm and 488 nm wavelength, respectively.^27^ This is 2.8 to 4.0 times shorter than the 800 nm film thickness. The contribution of Zn_3_P_2_ to the optical spectra is thus expected to dominate the contribution of the InP substrate. It is shown below that this is true, except for low-temperature photoluminescence spectroscopy. Topographical ridges can be seen crossing the cleaved cross-section and substrate. These are attributed to the strain at the interface and are sometimes observed after cleaving.

The unpolarized Raman spectrum of the film is shown in Fig. 1c. The spectrum is a clear fingerprint of the *D*_4h_ α-Zn_3_P_2_ lattice^28,29^ and does not show any contribution from the substrate. The peak positions of all phonons in the wavenumber range, determined experimentally in ref. 28, are shown with symbols under the spectrum.

The film is oriented with the *c*-axis perpendicular to the (100) InP substrate surface, as seen in the XRD pattern. The (004) peak of Zn_3_P_2_ is located at 2*θ* = 31.609 ± 0.026° in the pattern. Lattice parameter c calculated from the XRD pattern is equal to 11.67 ± 0.15 Å. This is about 2.4 ± 1.3% larger than the lattice parameters of bulk crystals.^30^ Considering uniaxial elongation of the crystal uniformly across the film, we estimate the other lattice parameter using Poisson's ratio *ν*, calculated from the elastic moduli in ref. 31. With 
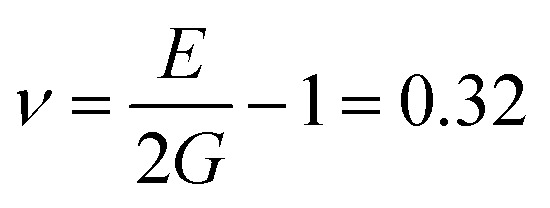
, where *E* and *G* are the Young and the shear moduli, respectively, the lattice parameter a is 8.02 ± 0.03 Å, or about 0.8 ± 0.4% smaller than measured in ref. 30. The lattice of this compound can be viewed as a stack of defected and slightly distorted anti-fluorite cubic cells. The reduced lattice parameter of the tetragonal lattice, *a*′, is related to the standard tetragonal lattice parameters *a*_t_ and *c*_t_ in the following way: 
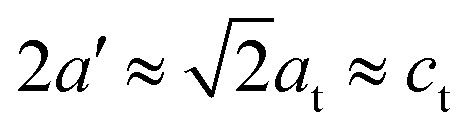
.^32^ Comparing the parameter *a*′ = 5.67 ± 0.02 Å, calculated from *a*_t_, with the lattice parameter of InP, 5.8687 Å, gives a lattice mismatch of 3.4 ± 0.4% between the Zn_3_P_2_ thin film and the InP substrate. The film is thus mostly relaxed overall, with a small uniaxial distortion along the *c*-axis. These values are also very similar to the lattice mismatch of ∼2% determined on other monocrystalline thin films by selected area electron diffraction (SAED) in transmission electron microscopy.^21^

**Fig. 2:**
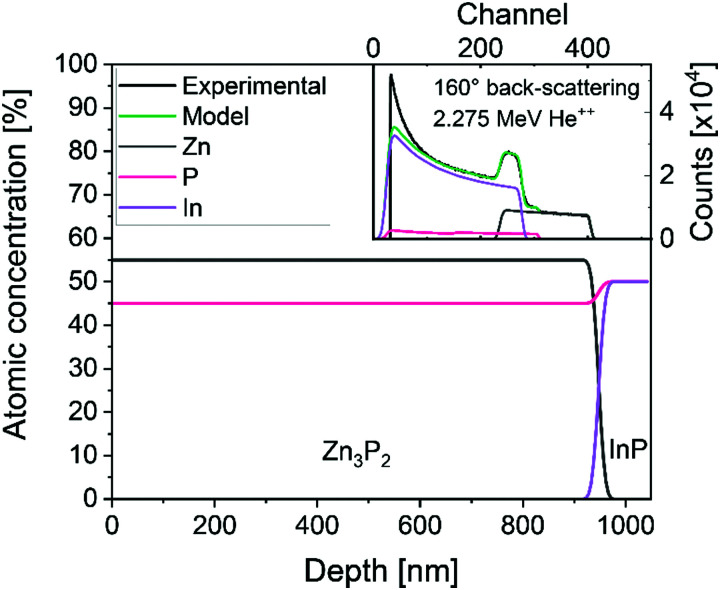
Representative composition along the depth of the sample, calculated from Rutherford back-scattering with 2.275 MeV He^++^. The inset shows the measured RBS spectrum in back-scattering (160°) and the fit of the spectrum. The spectrum at grazing exit (104°) is shown in the ESI† (Fig. S1).

The lattice of zinc phosphide can accommodate relatively large compositional variations away from stoichiometry while maintaining the crystalline structure of Zn_3_P_2_.^10^ The composition of our film across the depth, measured with Rutherford backscattering spectrometry (RBS), is shown in Fig. 2. The analysis of the data is consistent with a uniform composition of 55% of zinc and 45% of phosphorus, making the compound slightly phosphorus rich, compared to the 60 : 40 (Zn : P) composition of stoichiometric Zn_3_P_2_.

### 3.2. Photoluminescence spectroscopy

**Fig. 3:**
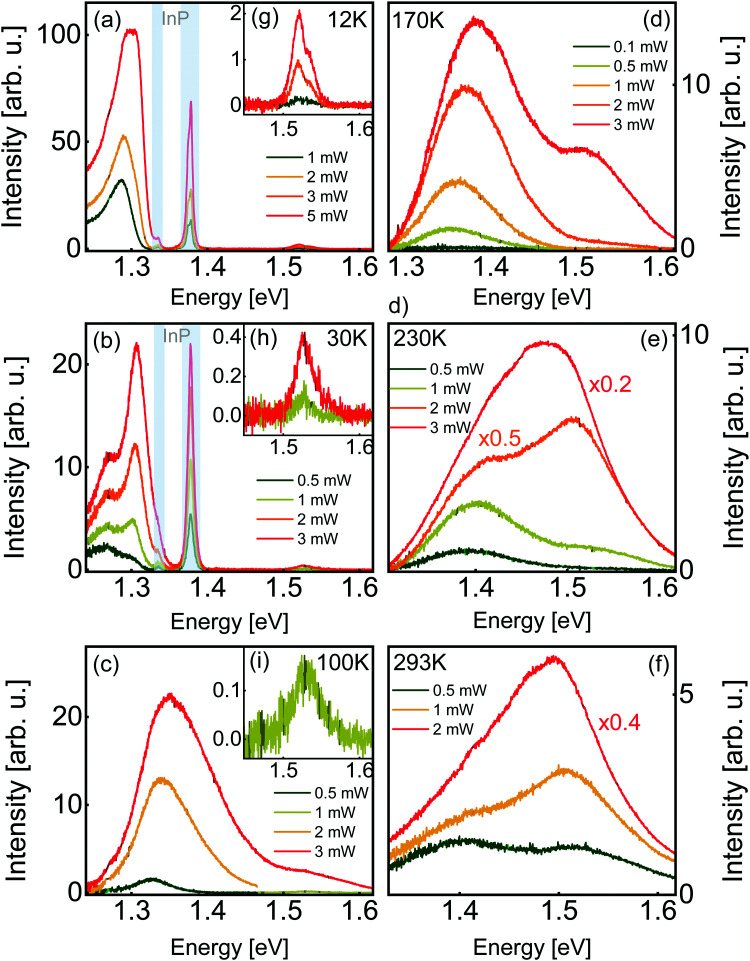
Photoluminescence spectra of a monocrystalline thin film acquired with a 488 nm laser at (a) 12 K, (b) 30 K, (c) 100 K, (d) 170 K, (e) 230 K and (f) 293 K. Insets (g–i) show magnified views of the peaks near 1.52 eV. Photoluminescence peaks assigned to the InP substrate are shown with grayed out areas. Shade differences in the graphs indicate that the data was altered for readability, as described in the main text.

Now that we have established the structural and compositional characteristics of our film, we turn to the optical properties by means of steady-state photoluminescence spectroscopy. The PL spectra of zinc phosphide, acquired with a 488 nm laser at different temperatures and excitation power densities, are shown in Fig. 3. The spectra are well described with two stable and consistent radiative contributions, one shifting significantly with temperature, and the other, at higher energies, with minimal shift. In addition to these consistent sources, the spectra also exhibit some irregular emissions only observed sporadically.

At 12 K and 30 K, two sets of peaks originating from the InP substrate can be observed, one series of defect-related peaks near 1.377 eV, and their phonon replicas 0.042 ± 0.002 eV lower.^33^ The positions of these peaks are indicated with grayed out areas. At temperatures of 100 K and higher, only emission from the film is observed. As discussed above, the light reaching the substrate is more than 6 orders of magnitude less intense than the excitation light contributing to the photoluminescence of zinc phosphide. We think that the observation of InP photoluminescence in our spectra is due to the diffusion of photoexcited charges from Zn_3_P_2_ to InP, where they would recombine. This hypothesis is supported by the fact that InP photoluminescence is not observed at higher temperatures, where carrier mobility is decreased by scattering effects and carrier recombination dynamics in Zn_3_P_2_ are altered.

At low temperatures (12 K to 30 K, Fig. 3a and b), the zinc phosphide spectrum consists of a set of very intense peaks in the 1.26–1.31 eV range, and a set of much weaker peaks in the 1.52–1.54 eV range, close to the direct bandgap energy of zinc phosphide.^34^ We designate these two sets of peaks as low-energy (LE) and high-energy (HE) peaks, respectively. Low-temperature spectra with intense peaks at energies of 1.41 eV and lower appear to be common in zinc phosphide.^6,20,35,36^

At higher temperatures (100 K to 293 K, Fig. 3c–f), the LE and HE peaks in the spectra are not only wider, making them more overlapped, but the broad LE peak is found at higher energies, centered in the range from 1.32 eV to 1.4 eV approximately. To the best of our knowledge, two other groups published near-room-temperature photoluminescence spectra of this material until today. The results compare relatively well. A peak near the bandgap energy of zinc phosphide is always observed, albeit sometimes without being accompanied by a second peak at lower energy.^6,37,38^

We have determined the nature of the transitions involved in the recombination processes for Zn_3_P_2_ by investigating the excitation intensity dependence of the PL spectra. Models of the different recombination processes in semiconductors indicate that when the incident excitation power (*P*) is varied over a range of at least two orders of magnitude, the relationship between the intensity of a single PL peak (*I*) is related to it by a power law of the form: *I* ∼ *P*^*k*^, where *k* is a coefficient depending on the nature of the transition.^39,40^ Values of *k* smaller than 1 are interpreted as a recombination involving localization of the carriers at defects with levels inside the band gap. On the other hand, values of *k* greater than 1 are related to free- and bound-exciton emission. Fig. S2 (ESI†) summarizes the *k* coefficient of the different observed peaks, wherever it can be estimated. All peaks observed in the PL spectra exhibit power dependency behavior with *k* > 1. Furthermore, some of the PL peaks observed in this study appear to have very high power coefficients (*k*), such as the peak at approximately 1.31 eV, which appears to have a coefficient *k* = 4.2 at 12 K. It must be noted that this peak is not clearly resolved, and it is currently unclear how many and exactly what kind of radiative electronic transitions contribute to the detected signal in the energy range near this peak. The apparent dramatic intensity increase could be caused by the interplay of multiple peaks.

#### 3.2.1. Low-energy radiative transitions

In the lowest-temperature spectra, acquired at 12 K and 30 K (Fig. 3a and b), the LE emission is composed of multiple peaks. The position of these peaks is quite consistent over the film, with some occasional shifts or new peaks close in energy. Fig. S3 in the ESI† shows different spectra acquired in the same conditions at different locations on a sample at 12 K.

At 30 K (Fig. 3b), the LE emission can be separated into two contributions, a symmetric peak near the high-energy edge and a wider symmetric peak at lower energies. The spectrum can be very well fitted with two Gaussians exhibiting regular and monotonous changes with increasing irradiance (Fig. S4, ESI†). At 12 K, the LE peaks on the low-energy side are more asymmetric and those on the high-energy side are more difficult to resolve (Fig. S5, ESI†). Nonetheless, the spectrum can be well described with an asymmetric and a symmetric peak. At 100 K, the LE and HE peaks are separated at low power, and overlap at higher powers. At low power, the LE peak is symmetric and becomes asymmetric at higher powers. Obtained peak properties from the fits are listed in Table 1. Both contributions shift to higher energy side with increasing laser power at rates in the range from 3 to 6 meV mW^−1^. These kinds of shifts, along with its asymmetric shape at low temperatures, may be related to band tail recombination mechanisms.^40,41^ High concentrations of randomly distributed charged defects can cause spatial potential fluctuations which in turn create tails in the electron and hole densities of states, at energies below the conduction band minimum, or above the valence band maximum. This might be the case in phosphorus-rich Zn_3_P_2_, due to the high concentration of phosphorus interstitials.

**Table 1:** Best fit parameters of the low-energy emission at low temperatures. (s) and (a) indicate that the corresponding peak is symmetric or asymmetric, respectively. All peaks shift towards higher energies with increasing power

Temp.	Note	First LE peak		Second LE peak	
		Position [eV]	Shift [meV mW^−1^]	Position [eV]	Shift [meV mW^−1^]
12 K		1.283 (a)	3.3	1.306–1.310 (s)	
30 K		1.262 (s)	5.6	1.299 (s)	3.5
100 K	Low power	1.326 (s)			
	High power	1.324 (a)	6.2		

At temperatures of 170 K and above, the LE and HE peaks overlap even at low power. They can be well described with one Gaussian describing each of the LE and HE peaks. Fig. S7 and S8 (ESI†) show the variation of the peak parameters with the laser power. Overall, as the temperature increases, the LE peaks shift to higher energies, while the HE peaks do not exhibit a noticeable shift in energy. In Fig. S2 (ESI†), the position of the LE (single or double) and HE peaks (single or averaged) are shown for the different temperatures. The reported peak positions are the result of least-square fitting with minimal numbers of Gaussians. The standard error on the best fit peak center is typically around a few meV.

There has long been a lack of consensus about the nature of the fundamental band transition of zinc phosphide,^6^ with claims of it being direct^42,43^ and other of it being indirect.^44,45^ Recently, valence electron energy-loss spectroscopy performed on Zn_3_P_2_ nanowires and spectroscopic ellipsometry on Zn_3_P_2_ monocrystalline thin films have reported Zn_3_P_2_ being a direct band gap semiconductor.^34^ An explanation to the apparent presence of a fleeting and unpredicted indirect fundamental band edge could be the existence of a defect band or band tails. The presence of an impurity band has already been shown to exist in Zn_3_As_2_, a II–V semiconductor structurally and electronically similar to Zn_3_P_2_.^46^ The critical defect concentration for the Mott transition in Zn_3_P_2_ and Zn_3_As_2_ are similar and may be around 2 × 10^16^ cm^−3^.^47^

One of the most common defects in zinc phosphide, and most especially in phosphorus-rich crystals, are phosphorus interstitials. These defects are commonly designated as the main reason for the nearly ubiquitous intrinsic p-type doping of zinc phosphide. When charged, these defects could facilitate the formation of defect bands or band tails due to their large delocalization.^13^ Such bands or degenerate doping have never been reported in zinc phosphide. However, given the very phosphorus-rich nature of our films and the aforementioned properties of the most common defects, the presence of an impurity band or band tails is a real possibility.

The existence of a defect band can explain the LE peak observed in these samples. Let us assume that the high concentration of defects, likely phosphorus interstitials acting as acceptors, creates an impurity band (IB) close to the valence band maximum (VBM), peaking in the center of the Brillouin Zone. Direct transitions between the conduction and the valence band are at the origin of the HE ∼1.5 eV peak in the photoluminescence spectrum, while indirect transitions between the conduction band and the impurity band result in the other main peak at lower energies. The HE peak does not shift significantly with temperature, while the LE peaks appear to blueshift with increasing temperature (Fig. S2, ESI†), at a rate of approximately 4.0 × 10^−4^ eV K^−1^, consistent in magnitude with band shifts reported in the literature for zinc phosphide.^20,42,48,49^ The VBM and the IB maximum would thus have different temperature dependences. This is consistent with some observations, where bands with temperature shifts of opposite signs have been observed experimentally in zinc phosphide.^20,49^

The energy levels involved in the observed LE PL emission may also have a yet undiscussed origin. It has recently been shown that rotated crystallites with interfaces free of dangling bonds can be formed during growth.^50^ DFT calculations have shown that the electronic bandgap at these interfaces is significantly decreased compared to the bulk, on the order of 0.1 eV smaller, and new localized acceptor levels are formed. The existence of these 2D defects also appears to have near to no additional energetical cost compared to their absence, though their dependence on temperature is still unknown. Additionally, the nanoscale growth process leading to the formation of these defects, *i.e.*, the growth and merger of differently-oriented Zn_3_P_2_ nuclei on (100)InP, is likely to have also occurred in the growth of the thin films discussed in this work. For these reasons, the presence of these defects in our thin films is likely, but their density and impact are undetermined. It is challenging to observe these rotated domain interfaces and additional work would be needed to quantify them in monocrystalline thin films and to experimentally verify their properties. Radiative recombination across the reduced bandgap at these interfaces could potentially be an origin of some or most sub-bandgap radiative transitions observed in this work.

#### 3.2.2. High-energy radiative transitions

Contrary to the LE peaks discussed in the previous section, the position of the HE peaks at about 1.5 eV is only weakly affected by temperature (Fig. S2, ESI†). At 12 K and 30 K, the HE peaks are about 50 to 100 times less intense than the LE peaks for all powers, but the intensities of the two sets of peaks become more balanced at higher temperatures. This kind of peak behavior suggest band to band transitions, between the valence and conduction bands. Furthermore, the position of the peaks matches well previously reported results for direct band gap of Zn_3_P_2_ at around 1.5 eV.^6,34^

At 12 K, the HE peak is a doublet (Fig. 3g). The lineshape is well fitted with two Gaussians with a separation of 18.1 meV to 18.6 meV and an intensity ratio of 3 to 4. At 30 K (Fig. 3h), the peaks are not well resolved, and can tentatively be assigned a splitting of 27 meV. At higher temperatures, the doublet cannot be resolved. The higher-energy peak is smaller than the lower-energy peak, like in Cd_3_P_2_.^51^ Contrary to the PL peak splittings reported until now for Zn_3_P_2_, this doublet cannot be due to a phonon replica since the lower intensity peak is at a higher energy. The measured splitting of the doublet is close to the crystal field splitting of zinc phosphide, reported to be around 0.02 eV^52^ to ∼0.04 eV.^42,43^ This is the first time the crystal field splitting of this semiconductor is observed in a photoluminescence spectrum, a testament to the exceptional crystalline quality of these structures.

### 3.3. Optical-pump terahertz-probe spectroscopy

To further characterize the temporal dynamics of photoexcited carriers, we studied the film with optical-pump terahertz-probe (OPTP) spectroscopy in reflection mode at room temperature. OPTP spectroscopy is sensitive to both radiative and non-radiative recombination processes, so the photoinduced response probes the temporal evolution of any conductive or polarizable charge species, including excitons, plasmons and free charges. The thin films were photoexcited at a center wavelength of 750 nm, corresponding to a photon energy of 1.65 eV. This photon energy is above the expected direct bandgap of Zn_3_P_2_, and hence should inject free electron–hole pairs within the thin film at room temperature.^44^ As the thickness of the thin film is greater than the absorption depth of Zn_3_P_2_ at 750 nm, the photoinduced THz response is dominated by the contribution from the Zn_3_P_2_ thin film with a negligible response from the InP substrate (see ESI†). Using the value of the absorption coefficient at this photoexcitation wavelength,^53^ the photoinduced carrier densities within the film were calculated to be between 4.9 × 10^17^ cm^−3^ and 5.17 × 10^18^ cm^−3^ for the fluence range.

**Fig. 4:**
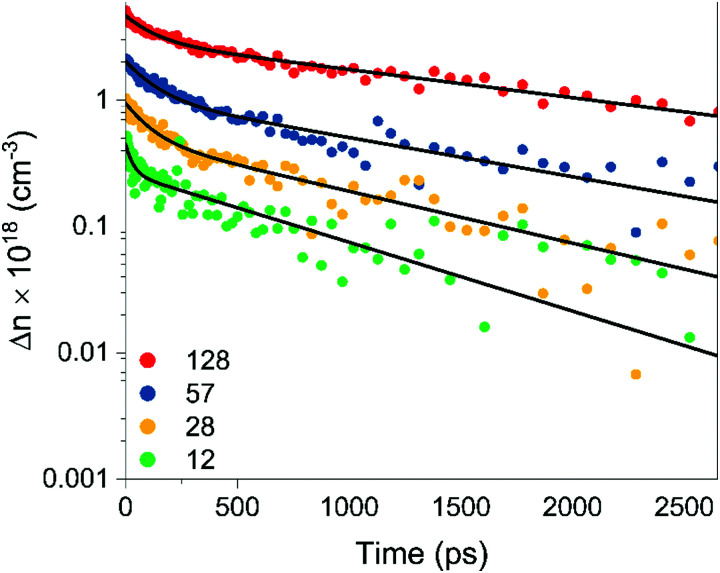
Biexponential decay of photoinduced charge carrier density as a function of time after photoexcitation measured by terahertz probe spectroscopy at four pump fluences.

Photoexcitation leads to a sharp rise in the THz photoconductivity followed by a biexponential decay on a nanosecond timescale. Fig. 4 shows the change in photoconductivity with time after photoexcitation at photoexcitation fluences of 12, 28, 57 and 128 μJ cm^−2^. Stronger pump fluences will photodope higher carrier densities into the conduction band. For the fluences used in this study, the peak photoconductivity and thereby excited carrier density is linearly related to the laser fluence (Fig. S10, ESI†). The decay curves displayed a biexponential behavior at all fluences, and the solid black lines in Fig. 4 show the fitted plots through the experimental data. The extracted characteristic decay constants of the biexponential fits are listed in Table 2.

**Table 2:** Characteristic decay times of the best biexponential fits to the carrier decay data

Pulse fluence [μJ cm^−2^]	*τ*_1_ [ns]	*τ*_2_ [ns]
128	0.12 ± 0.02	1.94 ± 0.05
57	0.12 ± 0.02	1.40 ± 0.05
28	0.10 ± 0.02	1.01 ± 0.05
12	0.03 ± 0.02	0.79 ± 0.05

At the timescales of this experiment, the carrier dynamics are dominated by a fast decay process (*τ*_1_) with a time constant of ∼0.1 ns, followed by a longer decay process (*τ*_2_) with a time constant of ∼1 ns. Both decay times increase slightly with increasing laser fluence and are likely attributable to surface and bulk recombination effects. The monoexponential behavior of the fast and slow decay components suggests that the recombination is dominated by monomolecular recombination. Both bulk band-edge and surface recombination pathways have been attributed to low-nanosecond range decay phenomena in zinc phosphide,^54,55^ and exciton recombination times are in the order of 0.1 ns.^56^

The native oxide of zinc phosphide passivates more than 90% of electrically active surface recombination states.^54^ Given that our samples have not been treated since their growth, the 0.1 ns fast decay component cannot be on account of surface recombination processes. We therefore attributed the 1 ns slow decay component instead to recombination through surface states. This is further supported by the increasing decay times with increasing fluence. Surface states become saturated at high carrier injections, slowing the recombination rate at the surface. On the other hand, the faster recombination pathway is attributed to band-edge recombination. This is consistent with the direct nature of the transition and the minor changes in recombination rates at higher fluences. Still, further studies are recommended to separate the effects of bulk and surface recombination more reliably and to identify the major bulk recombination pathways observable in OPTP spectroscopy.

## 4. Conclusion

In this work, we characterize the crystalline and photoluminescence properties of monocrystalline Zn_3_P_2_ thin films grown on (100) InP by molecular beam epitaxy. The films are phosphorus-rich and are oriented with the *c*-axis perpendicular to the substrate. The thin films exhibit two main radiative recombination pathways. A transition at approximately 1.52 eV is attributed to zone-center band-to-band electronic transitions. At 12 K and 30 K, the peak forms a doublet owed to the crystal field splitting of the tetragonal material. A second, lower-energy transition is observed at 1.3 eV to 1.4 eV and is attributed to a defect band or band tail transition. Phosphorus interstitials are likely at the origin of this band. Further studies should aim to more reliably separate surface and bulk effects with suitable surface treatments and to refine the identification of the specific defects involved in radiative recombination. The effect of the precise stoichiometry on the optical properties of thin films, in contrast to nanowires,^4,10^ should also be studied in the future.

## Conflicts of interest

There are no conflicts of interest to declare.

## Supplementary Material

MA-003-D1MA00922B-s001
