# Supplementary material for: Showcasing the optical properties of monocrystalline zinc phosphide thin films as an earth-abundant photovoltaic absorber
Source: Mater Adv. 2021 Dec 17;3(2):1295–303. doi: 10.1039/d1ma00922b (PMC8784961; doi:10.1039/d1ma00922b)
Supplement: MA-003-D1MA00922B-s001 [file MA-003-D1MA00922B-s001.pdf]

Electronic Supplementary Material (ESI) for Materials Advances.
This journal is © The Royal Society of Chemistry 2021

Supplementary information for

"Showcasing the optical properties of monocrystalline zinc phosphide thin films as an earth-abundant photovoltaic absorber"

Elias Z. Stutz†, Mahdi Zamani†, Djamshid A. Damry[꜔], Léa Buswell†, Rajrupa Paul†, Simon Escobar Steinvall†, Jean-Baptiste Leran†, Jessica L. Boland[꜔], Mirjana Dimitrievska†*, and Anna Fontcuberta i Morral†‡*

† Laboratory of Semiconductor Materials, Institute of Materials, Faculty of Engineering, École Polytechnique Fédérale de Lausanne, 1015 Lausanne, Switzerland
[꜔] Photon Science Institute, Department of Electrical and Electronic Engineering, University of Manchester, Alan Turing Building, Oxford Road, Manchester M13 9PL, United Kingdom
‡ Institute of Physics, Faculty of Basic Sciences, École Polytechnique Fédérale de Lausanne, 1015 Lausanne, Switzerland

Corresponding authors: mirjana.dimitrievska@epfl.ch; anna.fontcuberta-morral@epfl.ch

Additional RBS data

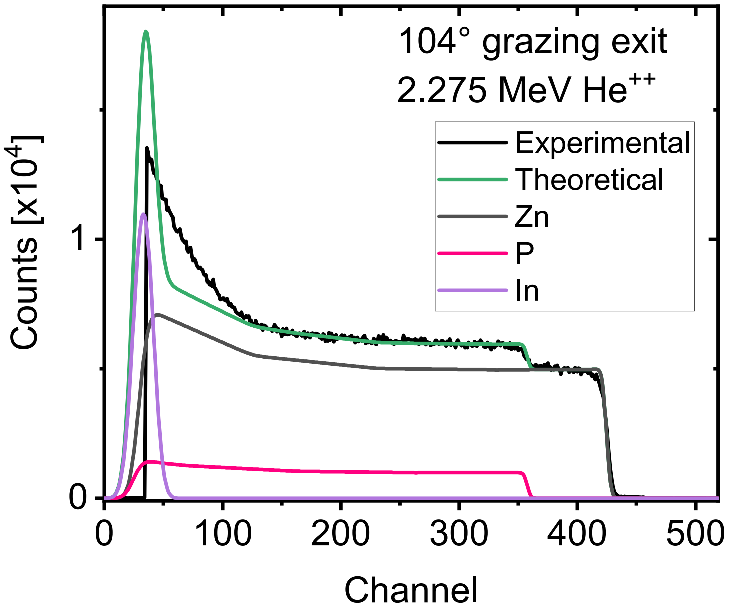

Figure S1: Rutherford backscattering spectrum acquired with a grazing exit detector at 104° from normally-impinging 2.275 MeV He^{++}. Elemental fits of the atoms in the Zn_3P_2 layer and InP substrate are shown.

Summary of photoluminescence peak properties

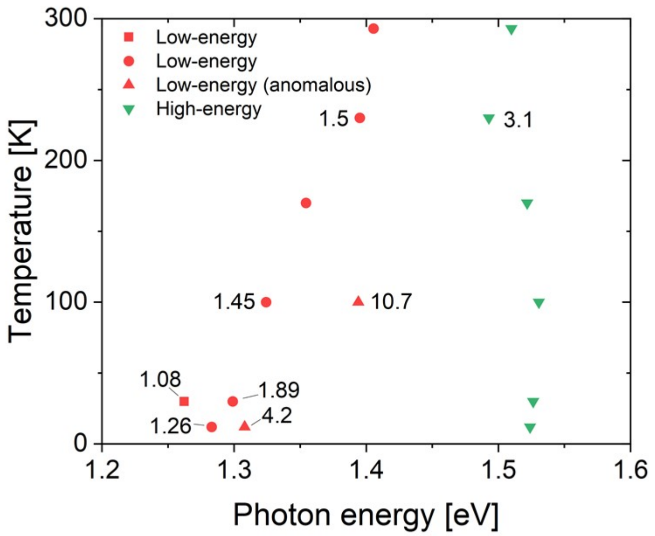

Figure S2: Peak center positions of the gaussians best fitting the data. For the high-energy peaks (green), at 1 mW. For the low-energy peaks (red) at 30 K and below, extrapolated to 0 mW. For the low-energy peaks (red) at 100 K and above, averaged over the range of measurements. Numerical values indicate the exponent of the $I=P^k$ relationship, linking the peak intensity (I) to the laser power (P).

Variability of the photoluminescence spectrum

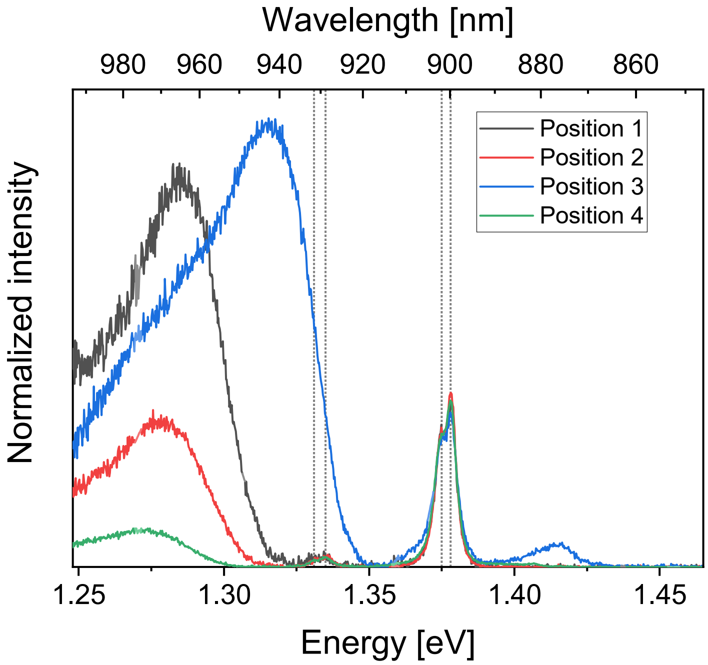

Figure S3: Photoluminescence spectra acquired with a 488 nm laser at 12 K at different positions. The defect peaks of the InP substrate and their phonon replicas are shown with vertical dotted lines. The spectra are normalized to the main InP peaks.

Fitting of the photoluminescence spectra

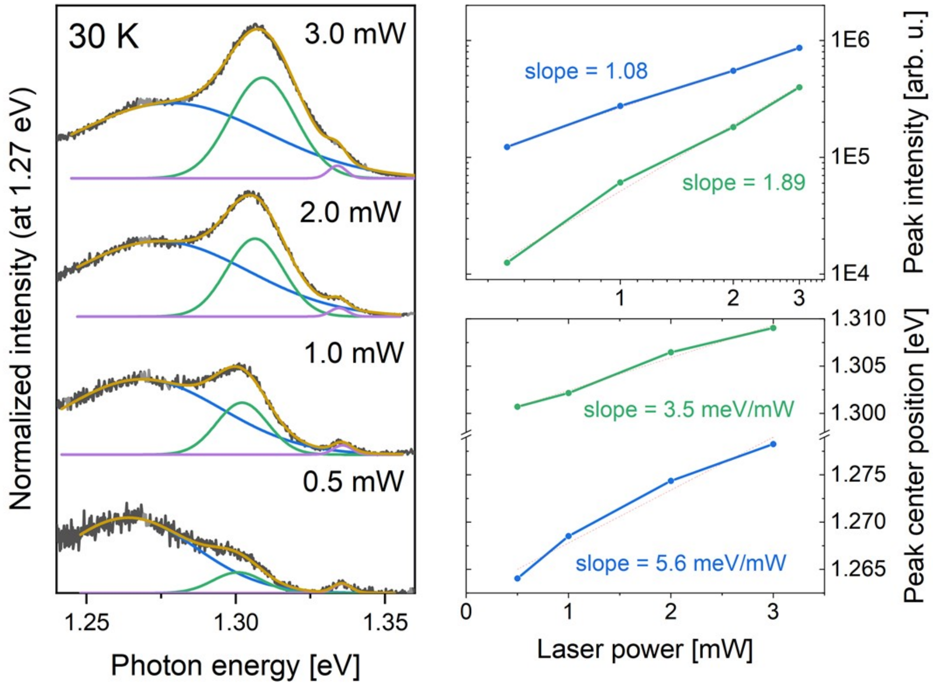

Figure S4: Detailed fitting of the sub-1.36 eV peaks in the spectra acquired at 30 K. The fits were carried out in the range from 1.24-1.25 eV to 1.36 eV with gaussians. No constraints were applied to the peaks besides their number, having a positive area, a baseline at y = 0 and the FWHM of the InP peak near 1.33 eV being constant.

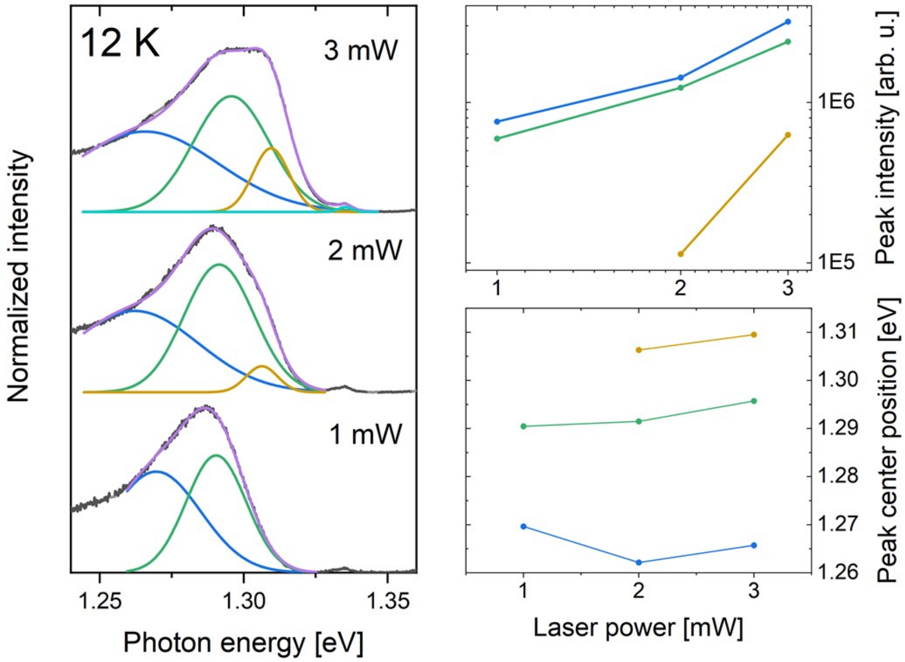

Figure S5: Detailed fitting of the sub-1.36 eV peaks in the spectra acquired at 12 K. The fits were carried out in the range from 1.24-1.25 eV to 1.36 eV with gaussians. No constraints were applied to the peaks besides their number, having a positive area and a baseline at y = 0.

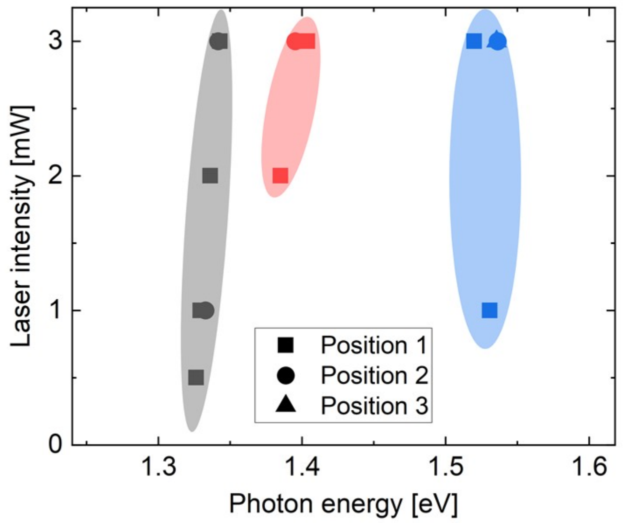

Figure S6: Best fit parameters (with two or three gaussians) of spectra acquired at 100 K. Data acquired at different positions on the same sample (data not shown).

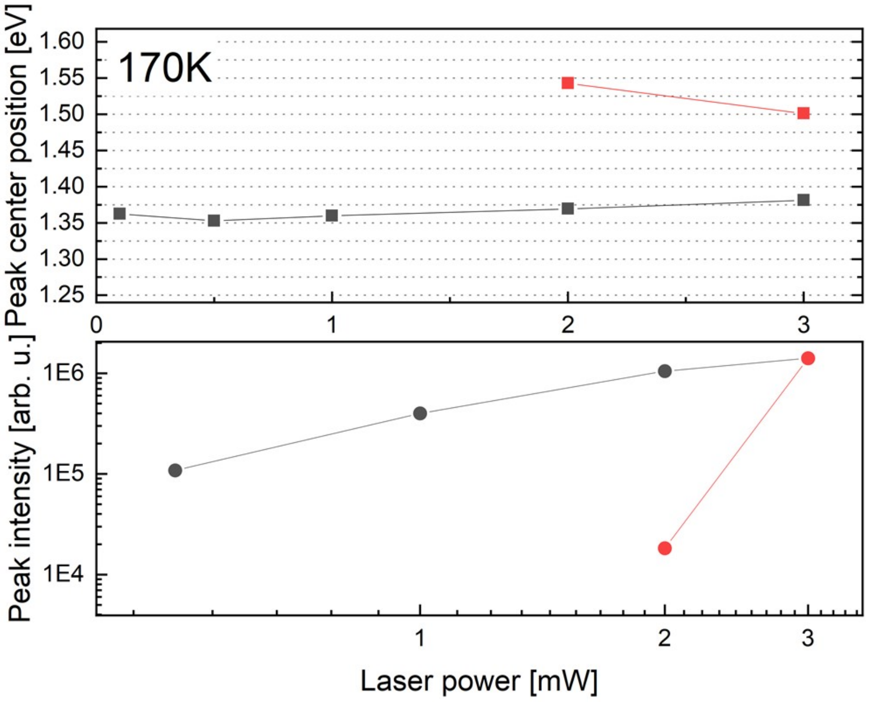

Figure S7: Best fit parameters (with two gaussians) of spectra acquired at 170 K. Top: peak center position. Bottom: peak intensity.

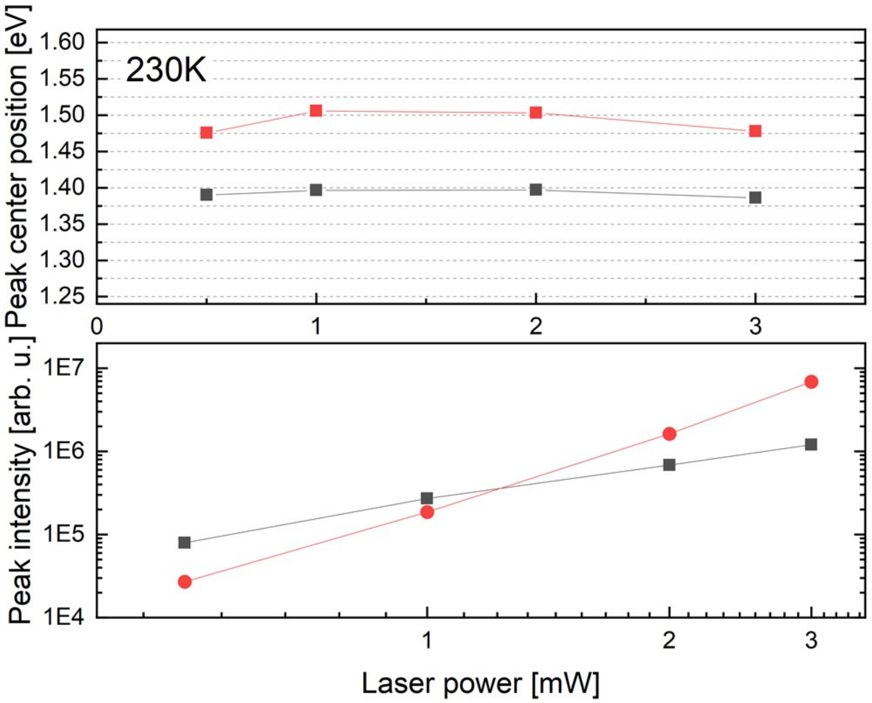

Figure S8: Best fit parameters (with two gaussians) of spectra acquired at 230 K. Top: peak center position. Bottom: peak intensity.

Optical pump terahertz probe data analysis – calculation of carrier density

For the reflection geometry used in the mansucript, the photoinduced change in THz reflected signal, ΔR is measured and referenced to the unphotoexcited THz reflected signal, R, as follows:

$$\frac{\Delta R}{R} = \frac{E_p - E_0}{E_0} = \frac{\Delta E_r}{E_r} \tag{1}$$

where E_p is the measured reflected THz electric field when the sample is photoexcited and E_0 is the THz electric field measured in equilibrium (i.e. unphotoexcited case). R is therefore the sample reflectivity without photoexcitation and is equal to:

$$R = \frac{1 - n_0}{1 + n_0} \tag{2}$$

where n_0 is the complex frequency-dependent refractive index of the unexcited sample at THz frequencies. This measured signal, $\frac{\Delta E_r}{E_r}$, is directly proportional to the effective photoinduced charge carrier density. The number of charge carriers induced by the pump beam is calculated using:

$$N = \varphi \frac{E\lambda}{hc}(1 - exp(-\alpha d)) \tag{3}$$

Where α is the absorption coefficient and d is the thickness of the thin film. E is the total energy and λ is the wavelength of the photoexcitation beam. φ is the ratio of free charge-carriers created per photon absorbed and is assumed to be unity ($\varphi = 1$), to provide a maximum value for the number of photoinduced charge carriers.

To calculate the effective photoinduced charge density in the thin film, the overlapping area between the optical pump beam and THz probe beam is also taken account. Both beams are assumed to have 2D Gaussian profiles, so that the effective overlap area is given by:

$$A_{eff} = 2\pi\left(w_{pump}^{2} + w_{THz}^{2}\right) \quad (4)$$

where w_{pump} and w_{THz} are the beam waists of the pump and THz beam respectively. The effective photoinduced charge carrier density is therefore given by:

$$n = \frac{N}{A_{eff}} \quad (5)$$

For this analysis, we used a measured value for the absorption coefficient of 1.85 x 10^4 cm^{-1} calculated from the extinction coefficient in reference[1]. This value is given for a photoexcitation with polarization perpendicular to the c-axis of the thin film, matching our experimental geometry. This value also coincides with other experimental studies[2,3].

Figure S9 shows the decay of the photoinduced charge carrier density as a function of time after photoexcitation.

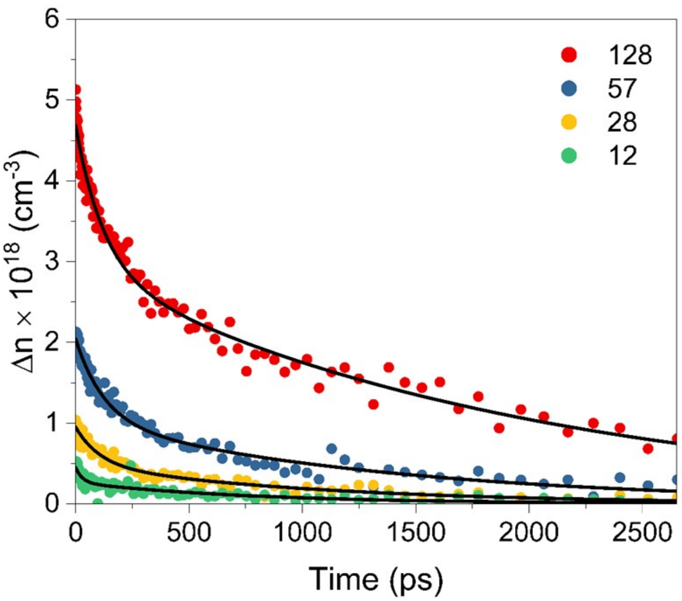

Figure S9: Decay of photoinduced carrier density as a function of time after photoexcitation.

For fluences of 128, 57, 28 and 12 μJ cm^{-2}, the calculated photoinduced carrier density values at time = 0 ps after photoexcitation were (5.17 , 2.3, 1.13, 0.49) x 10^{18} cm^{-3}.

Optical pump terahertz probe spectroscopy fitting

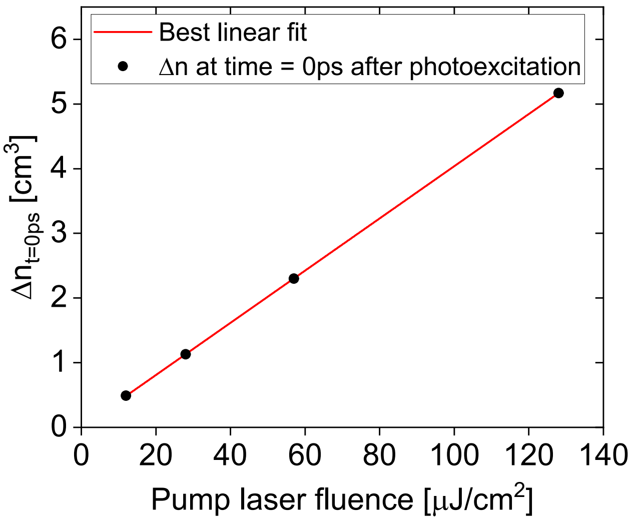

Figure S10: Maximum of the biexponential fit to the temporal OPTP data, versus pump fluence. The red curve is the best linear fit.

Optical pump terahertz probe data analysis – calculation of photoconductivity

To convert the transient photoinduced reflected THz signal into photoconductivity, the sample is modelled as a thin film on a thick bulk substrate. All multiple internal reflections of the probing THz pulse can therefore be separated by temporal windowing. The front face of the thin film is optically excited at a central wavelength of 750 nm and the thickness of the thin film is larger than the absorption depth of the material, so that the photoinduced THz response is dominated by the thin film with negligible contribution from the bulk substrate.

ΔE_r can be calculated via solving the wave equation, as demonstrated in ref. [4], so that the measured signal, ΔE_r is equal to:

$$\Delta E_r = -\frac{Z_0 t_1^2 a^2}{2n_1}\left[\int_0^L \exp(2ikx)\Delta\sigma(x)d + 2r_2\exp(2ikL)\int_0^L \Delta\sigma(x)dx + \left(r_2\exp(2ikL)\right)^2\int_0^L \exp \right. \quad (6)$$

where Z_0 is the vacuum impedance, $t_1=2n_1/(n_0+n_1)$ is the transmission coefficient of the Zn_3P_2 thin film and $r_2=(n_0-n_1)/(n_0+n_1)$ is the internal reflection coefficient at the interface between the thin film and InP substrate. For our sample geometry, n_1 is the refractive index of air ($n_1 = 1$), n_2 is the refractive index of InP ($n_2 = 3.2$); and n is the refractive index of the Zn_3P_2 thin film. The value for the refractive index of InP is an average of previously-reported values in the THz frequency range [5]. For the refractive index of Zn_3P_2, the static dielectric constant was used ($n = \sqrt{33}$), which is consistent with other previous studies in the THz range[1,2]. The parameter a takes into account the multiple internal refection of the THz probe inside the sample and is equal to:

$$a = \left[1 - r_1 r_2 exp(2ikL)\right]^{-1}. \quad (7)$$

No internal reflections of the THz probe beam were observed within the time-domain waveform, so a = 1. When the photoexcited part is much thinner than the sample thickness, the unexcited part of the sample can be considered as the substrate, so that $n_0 = n_2$ and $r_2 = 0$. The formula reduces to:

$$\frac{\Delta E_r}{E_r} = \frac{2Z_0 n_1}{n_0^2 - n_1^2} \int_0^L exp(2ikx)\Delta\sigma(x)dx \tag{8}$$

This assumption is also valid for our case of a thin film on a highly conductive substrate when the substrate refractive index is comparable to that of the thin film[6], as r_2 reduces rendering the second and third terms in equation 6 negligible. As the thin film is excited within the linear regime (see Figure S10), the complex photoconductivity is assumed to follow the Beer-Lambert absorption law as a result of an exponential excitation profile:

$$\Delta\sigma(x) = \Delta\sigma_s exp(-\alpha x) \tag{9}$$

Where $\Delta\sigma_s$ is the photoconductivity at the surface of the thin film. Therefore, the measured signal is equal to:

$$\frac{\Delta E_r}{E_r} = \frac{2Z_0 n_1 \Delta\sigma_s}{n_0^2 - 1} \frac{1}{\alpha} \frac{1}{1 - \frac{2i\omega n_o}{\alpha c}} \tag{10}$$

The figure below shows the calculated frequency-averaged photoconductivity as a function of time after photoexcitation:

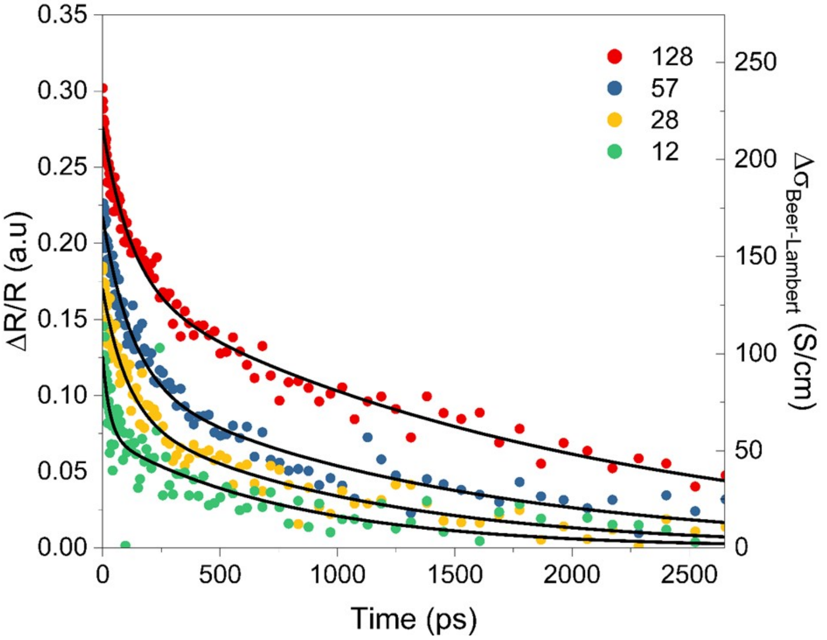

Figure S11: Frequency-averaged photoconductivity of the Zn_3P_2 thin film and the measured ΔR/R signal as a function of time after photoexcitation.

These values coincide with previously-reported values of > 100 S/cm for photoexcitation above 1.55 eV.

Photoconductivity decay within the thin film

As the OPTP spectroscopic measurements were peformed in reflection geometry, the observed photoinduced THz response is dominated by the photoconductivity at the surface of the Zn_3P_2 thin film. To illustrate this point, we model the expected photoconductvity from the InP substrate in our experimental configuration. Given the value of absorption coefficient used for the Zn_3P_2, we calculate that 22 % of the intensity of photoexcited excitation will reach the InP susbtrate. Taking into account absorption within the InP [5], this equates to an effective photoinduced carrier density on the order of 1 x 10^{15} cm^{-3}, which is three orders of magnitude lower than the photoinduced carrier density in the Zn_3P_2 thin film and comparable to the equilibrium carrier concentration of InP.

The photoconductivity was calculated for this carrier density (n = 1 x 10^{15} cm^{-3}) using a Drude response. The intrinsic carrier concentration was taken as n_0 = 3.79 x 10^{15} cm^{-3}; the scattering time, τ = 0.21 ps; and the electron effective mass as m_e^* = 0.08 m_e [5]. Figure S11 shows the calculated photoconductivity response.

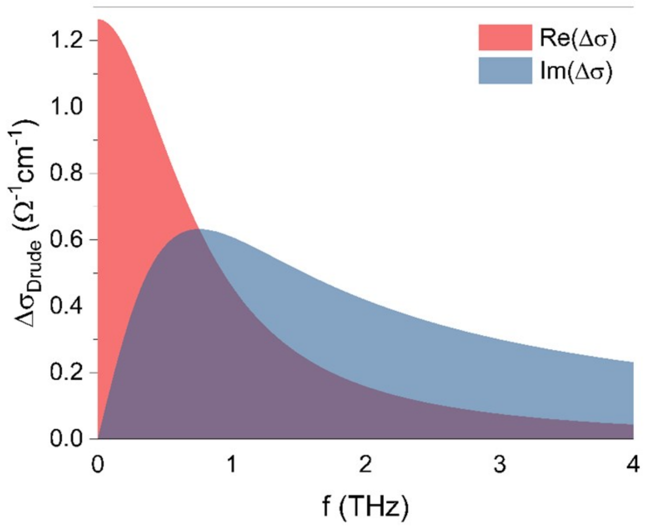

Figure S12: The calculated photoconductivity spectrum of the InP susbstrate when 22% of the photoexcited light intensity reaches its surface.

The magnitude of the photoconductivity for the InP susbtrate only reaches a maximum of ~1.2 S/cm, which is significantly smaller than the photoconductivity of the Zn_3P_2 thin film and below the noise floor of our measurement. The measured photoinduced response can therefore be attributed solely to the Zn_3P_2 thin film.

Using the expression for the Beer-Lambert law, Figure S12 shows the decay of the photoconductivity as a function of distance within the film.

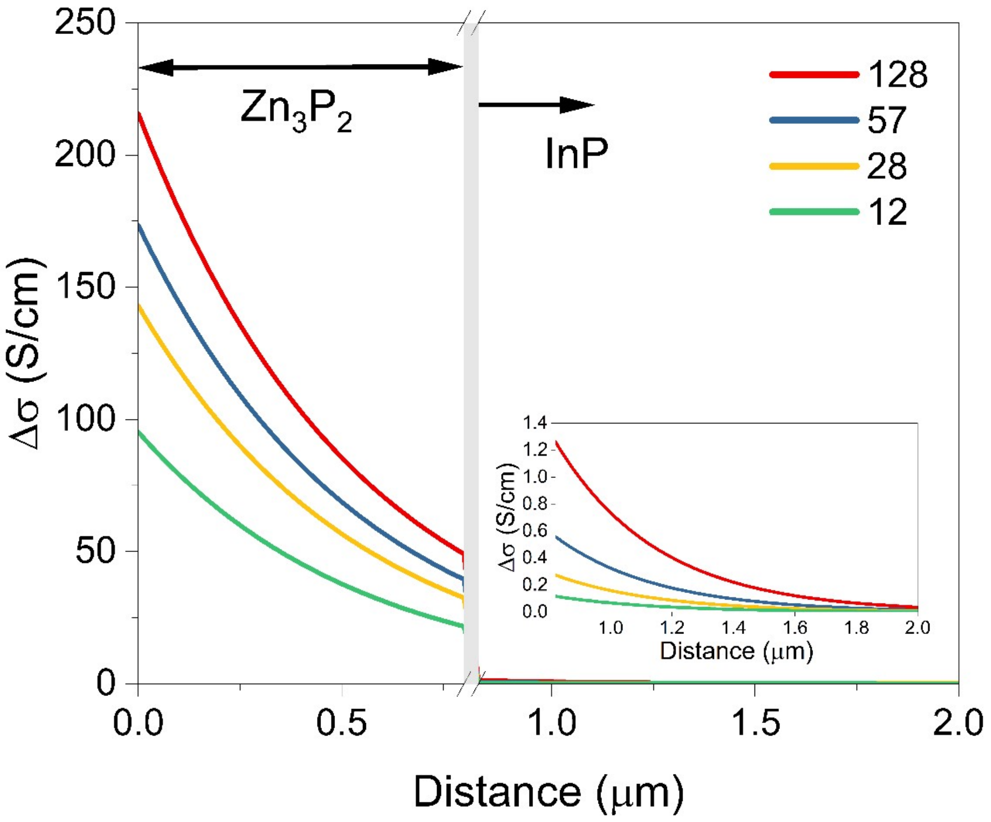

Figure S12: Change in photoconductivity as a function of distance within the Zn_3P_2 thin film and InP susbtrate.

The photoconductivity decays exponentially into the Zn_3P_2 thin film until it reaches the film thickness. At the film thickness, only 12% of the light intensity is absorbed by the InP substrate, leading to a reduced photoconductivity <1.2 S/cm. We therefore attribute the induced THz response solely due to the Zn_3P_2 thin film, with the dominant contribution from the surface of the thin film.

References

(1) Misiewicz, J.; Jezierski, K. Zinc Phosphide (Zn3P2). In *Handbook of Optical Constants of Solids*; Palik, E. D., Ed.; Academic Press, 1997; Vol. 2, pp 609–635. https://doi.org/10.1016/b978-012544415-6.50122-9.

(2) Zinc Phosphide (Zn3P2) Optical Properties, Dielectric Constant. In *Non-Tetrahedrally Bonded Elements and Binary Compounds I*; Madelung, O., Rössler, U., Schulz, M., Eds.; Springer-Verlag Berlin Heidelberg, 1998. https://doi.org/10.1007/10681727_210.

(3) Pawlikowski, J. M. Zn3P2 as Infrared-to-Ultraviolet Photoconverter. *Infrared Phys.* **1988**, *28* (3), 177–182. https://doi.org/10.1016/0020-0891(88)90007-3.

(4) D'Angelo, F.; Němec, H.; Parekh, S. H.; Kužel, P.; Bonn, M.; Turchinovich, D. Self-Referenced Ultra-Broadband Transient Terahertz Spectroscopy Using Air-Photonics. *Opt. Express* **2016**, *24* (9), 10157. https://doi.org/10.1364/oe.24.010157.

(5) Zhang, C.; Jin, B.; Chen, J.; Wu, P.; Tonouchi, M. Noncontact Evaluation of Nondoped InP Wafers

by Terahertz Time-Domain Spectroscopy. *J. Opt. Soc. Am. B* **2009**, *26* (9), A1–A5. https://doi.org/10.1364/JOSAB.26.0000A1.

(6) Hempel, H.; Unold, T.; Eichberger, R. Measurement of Charge Carrier Mobilities in Thin Films on Metal Substrates by Reflection Time Resolved Terahertz Spectroscopy. *Opt. Express* **2017**, *25* (15), 17227. https://doi.org/10.1364/oe.25.017227.
